# Book Notices

**Published:** 1868-03-15

**Authors:** 


### *BOOK NOTICES.*

---

THE DIAGNOSIS, PATHOLOGY AND TREATMENT OF DISEASES OF WOMEN; Including the Diagnosis of Pregnancy. By GRAILY HEWITT, M.D., London, F.R.C.P., Professor of Midwifery and Diseases of Women, University College, etc., etc. First American, from the second London Edition, Revised and Enlarged, with one hundred and sixteen Illustrations. Philadelphia: Lindsay & Blakiston, 1868. Pp. 707. For sale in Chicago by W. B. Keen & Co. $6.

The first London edition of this book was received with remarkable favor by English practitioners. The present edition has been thoroughly revised, and in great part re-written. In connection with the treatment, special attention has been paid to pathology. Sixty of the illustrations are original. Without being excessively voluminous, the book may fairly claim to present a very complete review of the important subjects comprised under its title. It is clear and compact, and the JOURNAL cordially recommends it to the perusal of the profession.

PROCEEDINGS OF THE AMERICAN PHARMACEUTICAL ASSOCIATION, at the Fifteenth Annual Meeting, held at New York City, September, 1868. Also the Constitution and Roll of Members. Pp. 453. From John M. Maisch, Penn., Sec. Am. Pharm. Association.

Copies of this important volume can be obtained in Chicago by addressing or calling upon our friend E. H. Sargent, corner State and Randolph streets, who has extra copies for sale for the benefit of the Association. It is a record of great industry, and should be in the possession of every druggist; while physicians will find in it much matter of reference which they can not readily obtain elsewhere — such as investigation of active principles, new remedies, improved modes of preparation or dispensing, etc., etc.

CONSUMPTION in New England and Elsewhere; or, Soil-Moisture one of its Chief Causes. Address delivered before the Massachusetts Medical Society. By Henry I. Bowditch, M.D. Second Edition. Boston: David Clapp & Son. 1868. From the author.

Dr. Bowditch's views are at present widely known, and their dissemination has gone far towards establishing the true philosophy of management of phthisical cases. The all-important thing is to have the patient change his climate — not to one cold or hot, but where, with moderate thermometric changes, the air is uniformly dry. No better climate can be found than the interior table-lands of this continent. If anything will "cure consumption," it is the climate where the hunters do not have to smoke or salt their venison or buffalo meat, but where it dessicates speedily, without any taint of putrefaction.

REPORT Presented to the Ninth Annual Meeting of the New Sydenham Society, held at Dublin, Aug. 10th, 1867; with List of Works Published, and other Information. Richard J. Dunglison, M.D., Hon. Local Secretary, 116 Girard st., Philadelphia.

The annual subscription to this useful society is $7.50, in advance, (the duty, etc., payable on arrival of the volumes, amounting to about $2.50 additional.)

Mr. S. R. Wells, editor Phrenological Journal, has published:

THE GOOD MAN'S LEGACY; an excellent Sermon, by Rev. SAMUEL OSGOOD, D.D. With Portrait and Sketch of Dr. Richard Rothe, of Heidelberg. Price, 25 cents.

CONSUMPTION; Its Cause, and Cure by the Swedish Movement. With Illustrations and Directions for Home Application. By DAVID WARK, M.D. Price, 30 cents.

EDUCATION OF THE HEART; The Necessity of Moral Culture for Human Happiness. By Hon. SCHUYLER COLFAX. Sent post-paid for 10 cents. Address the Publisher, 389 Broadway, New York.

CONSTITUTION, By-Laws, and Code of Ethics, of the Chicago College of Pharmacy; with a List of Officers and Members. Organized and Incorporated, Sept. 5th, 1859.

From an accompanying slip, we learn that the College contemplates the institution of a course of lectures on chemistry, pharmacy, materia medica, and the collateral sciences, commencing October next. Thos. Whitfield, 281 State street, is chairman of the committee having the matter in charge.

